# Intraventricular glioneuronal tumor with disseminated lesions at diagnosis - a case report -

**DOI:** 10.1186/1746-1596-6-119

**Published:** 2011-12-06

**Authors:** Hirohito Yano, Noriyuki Nakayama, Yoshinobu Hirose, Naoyuki Ohe, Jun Shinoda, Shin-ichi Yoshimura, Toru Iwama

**Affiliations:** 1 Department of Neurosurgery, Gifu University Graduate School of Medicine, Gifu, Japan; 2 Pathology Division, Gifu University Hospital, Gifu, Japan; 3 Chubu Medical Center for Prolonged Traumatic Brain Dysfunction, Department of Neurosurgery, Kizawa Memorial Hospital, Minokamo, Japan

**Keywords:** dissemination, glioneuronal tumor, immunohistochemistry, intensity-modulated radiation therapy, temozolomide

## Abstract

A 55-year-old man presented with a large tumor in his lateral ventricles. Magnetic resonance imaging revealed disseminated lesions in the third and fourth ventricles at the time of diagnosis. The patient underwent a partial removal of the tumor in the lateral ventricles. Histologically, the surgical specimens showed glioneuronal differentiation with ganglion or ganglioid cells, Rosenthal fibers, oligodendroglia-like honeycomb appearances, a spongy pattern, perivascular pseudorosettes, and many hyalinized blood vessels. Papillary structure was not observed. The neuronal component showed a moderately high labeling index of Ki-67/MIB-1. We diagnosed this tumor as atypical intraventricular glioneuronal tumor. The disseminated lesions disappeared after chemoradiation therapy with temozolomide, and the residual tumors in the lateral ventricles remained stable for 3 years after the surgery. We discuss the pathological diagnosis, therapy and clinical course with review of the literatures.

## Background

Many types of brain tumors originate in the ventricles. For tumors with neuronal differentiation that occupy the anterior central part of the lateral ventricles in adults, central neurocytoma is typically the diagnosis [1]. However, new entities of tumors with neuronal differentiation, which are rare, have been found in this region. Herein, we report an unusual case of a patient who had an atypical tumor with glioneuronal differentiation that arose from the lateral ventricles and that was already accompanied with intraventricular disseminations at the time of diagnosis. We discuss the rationale for the pathological diagnosis on the basis of the immunohistochemistry, the pathophysiology of the dissemination in the early clinical stages, and possible therapies for this tumor with a review of the literature.

## Case presentation

**Figure 1:**
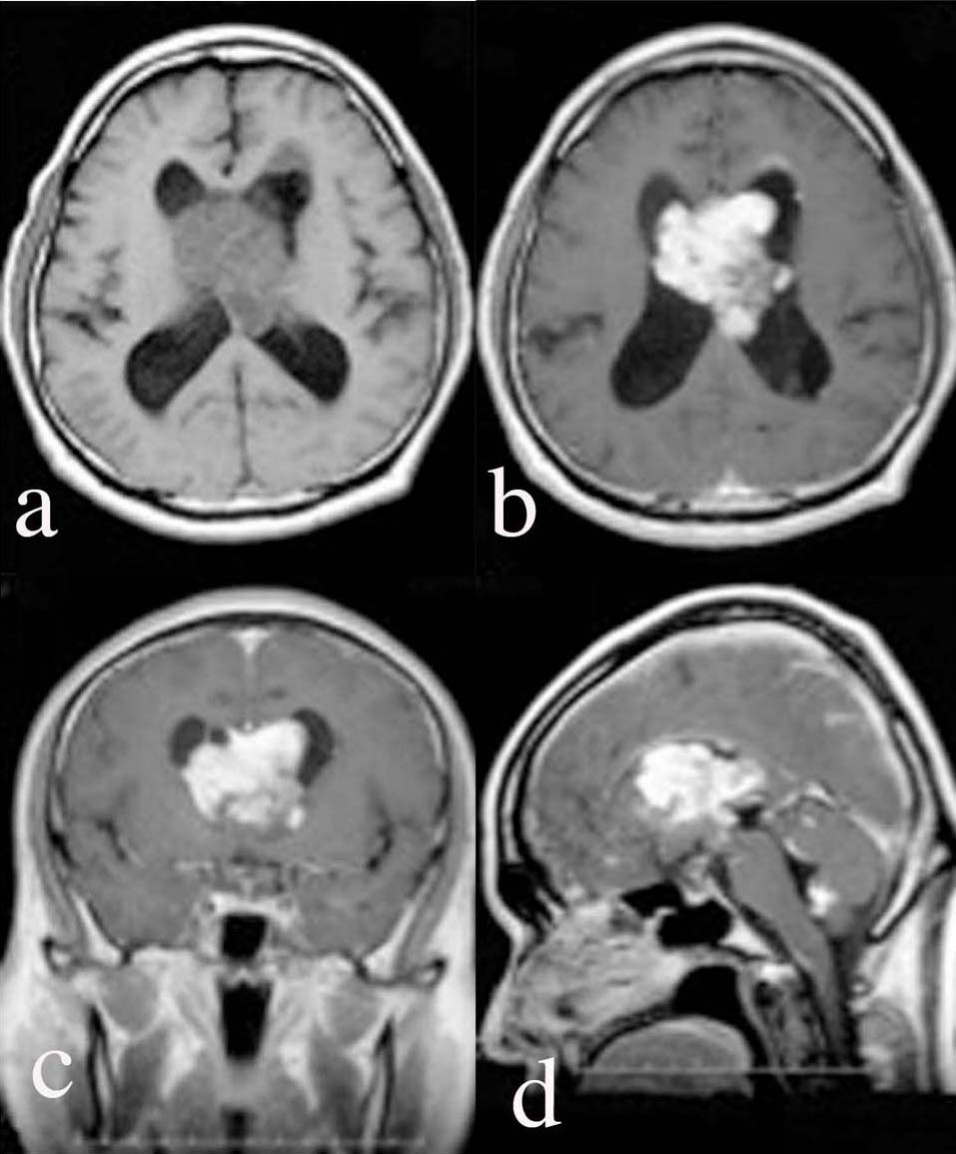
**Magnetic resonance imaging (MRI) on admission**. MRI [a: T1 plain-weighted image, b-d: Gadolinium-diethylenetriaminepentaacetic acid (Gd-DTPA)-enhanced images] scans showed a mass lesion in the lateral ventricles, the third ventricle, and the fourth ventricle. The mass lesion was enhanced with Gd-DTPA.

A 55-year-old man had a 6-month history of memory loss and stagger. He was admitted to our hospital with the diagnosis of an intraventricular tumor. Upon admission, his consciousness level was clear without focal neurological deficits. His Hasegawa's dementia scale (HDR) score had deteriorated to 12/30 points due to his recent memory disturbance. He did not show any symptoms of increased intracranial pressure. His hematological tests and physiological function tests were in the normal range. A computed tomography (CT) scan showed a large mass lesion of about 6 cm in diameter in the central part of the lateral ventricles. No calcification was shown in the lesion. Magnetic resonance imaging (MRI) revealed that the lesions had spread to the third and fourth ventricles (Figure 1a-d). MRI did not show any spinal lesions. The tumor occupying the lateral ventricle showed a low-signal intensity on T1-weighted images (WI), a high-signal intensity on T2-WI and fluid-attenuated inversion recovery (FLAIR) images, and the tumor was enhanced homogeneously with gadolinium (Gd). Positron emission tomography showed a homogeneously high uptake of ^11^C-methionine and a moderate uptake of ^18^F-fluorodeoxyglucose. A carotid angiography showed mild tumor staining. These findings led us to the primary diagnosis of central neurocytoma. The patient underwent a subtotal removal of the tumor by a frontal interhemispheric transcallosal approach. Intraoperatively, the tumor appeared ashy in color. It was soft superficially and elastic and hard internally. The tumor was adhered strongly to the septum pellucidum and moderately to the walls of the anterior horns and the bodies of the lateral ventricles. The choroidal arteries fed the tumor. Incidentally, the body of the right fornix was injured in order to remove the tumor extending into the third ventricle. Postoperative MRI showed that the tumor remained in the tip of the left anterior horn and the fourth ventricle. After the operation, the patient's memory disturbance worsened, and his postoperative HDS-R scale was 10/30.

### Pathological examination

Formalin-fixed, paraffin-embedded tissue sections were examined with hematoxylin-eosin (HE) staining and immunohistochemistry. The primary antibodies and their dilution with buffer were as follows: rabbit polyclonal anti-olig2 antibody (1:200; Millipore, Temecula, CA), polyclonal anti-vimentin antibody (1:200; Dako, Glostrup, Denmark), mouse monoclonal anti-glial fibrillary acidic protein (GFAP) antibody (1:500; Dako), mouse monoclonal anti-synaptophysin (Syn) antibody (1:50; Millipore), mouse monoclonal anti-tubulin, βIII isoform (TuJ1) antibody (1:200; Millipore), monoclonal anti-neuronal nuclear antigen (Neu-N) antibody (1:100; Millipore), monoclonal anti-microtubule associated protein-2 (MAP-2) antibody (1:100; Millipore), monoclonal anti-neuron-specific enolase (NSE) and monoclonal anti-Ki-67/MIB-1 antibody (1:50; Dako). Antigen retrieval using an autoclave (121°C, 15 min) was performed for all antibodies. An Envision kit (Dako) was used as a source of secondary antibodies conjugated to dextran polymer and hydrogen peroxidase, and 3,3-diaminobenzidine was used as the chromogen.

### Pathological findings

**Figure 2:**
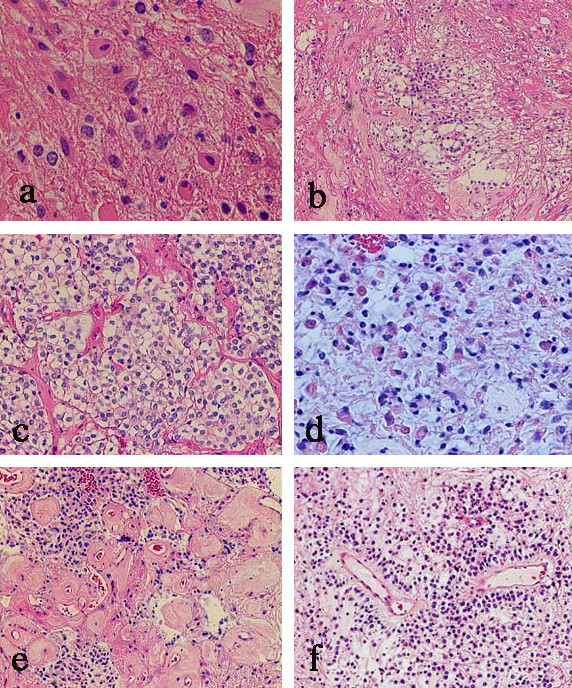
**Photomicrographs of hematoxylin-eosin staining**. (a) Several ganglioid cells with moderately abundant cytoplasm were observed. (b) A spongy pattern, (c) an oligodendroglial honeycomb pattern, (d) distinctive minigemistocytes, (e) well-developed hyalinized blood vessels, and (f) perivascular pseudorosette pattern. (a: ×400; b, e, f: ×100; c, d: ×200).

HE slides exhibited a variety of glioneuronal tumors. Some ganglions or ganglioid cells were observed with a background of neuropil (Figure 2a). Spongy patterns with many bipolar cells were also observed (Figure 2b). Oligodendroglioma-like honeycomb appearances were seen in part of the tumor (Figure 2c). Part of the tumor included a cluster of minigemistocytes (Figure 2d). Many hyalinized blood vessels were very visible, and a small amount of neoplastic blood vessels and endothelial proliferation was observed (Figure 2e). These findings exhibited some resemblance to pilocytic astrocytomas (PA), except for the presence of the ganglion cells. In some areas, cells with small round nuclei that surrounded and enclosed the vessels mimicked perivascular pseudorosettes (Figure 2f). Mitotic figures and necrotic areas were observed infrequently.

**Figure 3:**
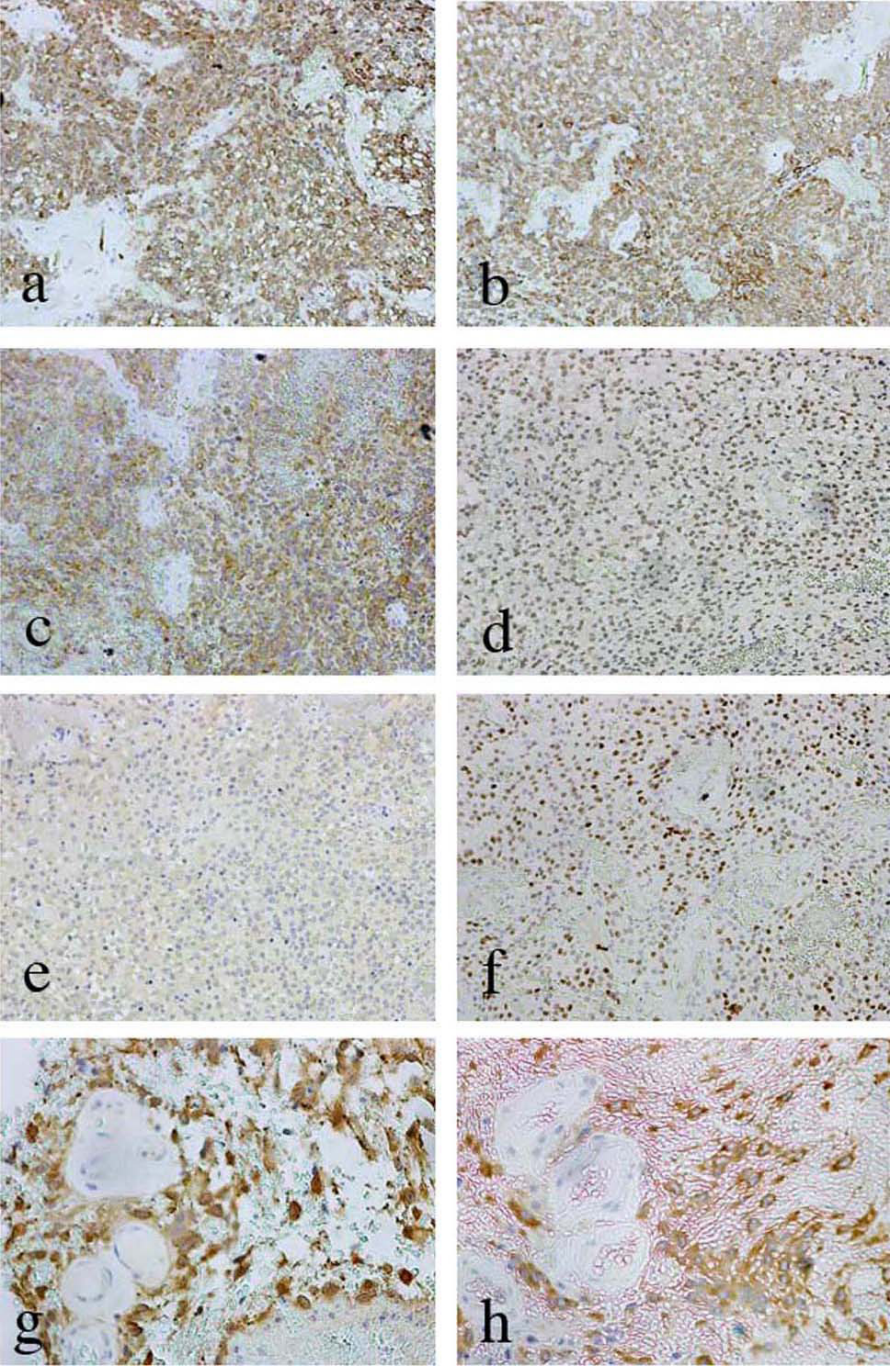
**Photomicrographs showing immunohistochemistry for neuronal markers**. (a) neuron-specific enolase (NSE), (b) microtubule-associated protein-2 (MAP-2), (c) Synaptophysin, (d) neuronal nuclear antigen (NeuN), (e) tubulin, βIII isoform (TuJ1). The markers in a-d were diffusely positive in the part of the tumor with neuronal differentiation. However, TuJ1 was only weakly positive there. Olig2-positive cells were also observed in the same area (f). The cells comprising the perivascular pseudorosette were strongly positive for NSE (g) and MAP-2 (h), a-f: ×100; g and h: ×200.

The results of the immunohistochemistry are shown in Table 1. The specimens exhibited both glial components and neuronal components. The glial components were strongly positive for GFAP and vimentin. In addition, the minigemistocytes and the Rosenthal fibers were strongly positive for GFAP. Syn- or MAP-2-positive cells were not observed. The neuronal components included cells that had moderately uniform oval nuclei. These cells were negative for GFAP and vimentin. Only the endothelial cells and reactive astrocytes were positive for GFAP and vimentin. The cells with oval nuclei were strongly positive for NSE, MAP-2, Syn, NeuN, Olig2, and they were mildly positive for TuJ1 (Figure 3a-f). The cells near the perivascular pseudorosettes were strongly positive for NSE and MAP-2 (Figure 3g, h). Some scattered cells were positive for TuJ1 and Olig2 around the perivascular pseudorosettes. MIB-1 labeling indices were 0.8% in the glial component and 8% in the neuronal component. These pathological findings resulted in the diagnosis of an intraventricular tumor with atypical glioneuronal differentiation.

**Table 1:** Results of the immunohistochemistry

	Glial components	Neuronal components
GFAP	++	+/-
Vimentin	++	+/-
NSE	+	++
MAP-2	-	++
NeuN	+/-	++
TuJ1	+	+
Olig2	-	++
Syn	-	++
Mib-1 LI	0.8%	8%

GFAP: glial fibrillary astrocytic protein, NSE: neuron-specific enolase, MAP-2: microtubule-associated protein 2, NeuN: neuronal nuclear antigen, TuJ1: anti-tubulin, βIII isoform, Syn: synaptophysin, LI: labeling index, -: negative, +/-: slight, +: mild, ++: strong.

### Postoperative course

For the residual tumor, the patient underwent intensity-modulated radiation therapy (IMRT) using helical tomotherapy, which consisted of 30 fractions and a median dose of 30.6 Gy. Temozolomide (TMZ: 150 mg/m^2^) was orally administered as a concomitant and adjuvant therapy following radiation therapy. Twelve months after the surgery, MRI with Gd revealed that the disseminated lesions in the lateral ventricles and fourth ventricle had disappeared. A small residual nodule remained in the tip of the left anterior horn. MRI with Gd showed that the residuum had remained stable for 30 months after the surgery. His memory disturbance gradually subsided, and his HDS-R scale improved to 25/30.

## Discussion

The tumor in this case exhibited components that showed perinuclear halos, which were suggestive of central neurocytoma. However, the tumor differed from central neurocytoma because it exhibited a small number of ganglion cells or ganglioid cells that were immunohistochemically positive for multiple neuronal markers. Additionally, the glial components observed in this tumor are uncommon in central neurocytomas. Although there is a possibility that these glial components might be trapped cell in the tumor, we regarded them as neoplastic cells because these components in the present case were too much as trapped cells. On the contrary, neuronal differentiation was not sufficient for central neurocytoma. Furthermore, the spongy pattern and abundant hyalinized vessels observed in this tumor were not consistent with the histological features of central neurocytoma. Hyalinized vessels are more frequently observed in papillary glioneuronal tumors (PGNT). However, the tumor of this case had less papillary structures and less ganglion cells than PGNTs, in which tumor cells are typically arranged around vessels, forming pseudopapillary structures [2].

Spongy patterns, Rosenthal fibers, hyalinized vessels, and honeycomb-like components have often been associated with PA [3]. However, PA is usually found in younger people. Additionally, the lateral ventricle is not a typical site for PA, which usually derives from the optic chiasm, hypothalamus, thalamus, basal ganglia, cerebral hemispheres, or cerebellar hemispheres [4].

Along with PGNTs, rosette-forming glioneuronal tumors of the fourth ventricle (RGNT) were recently listed in the fourth edition of the World Health Organization Classification of Tumours of the Central Nervous System [4,5]. It is notewothy that RGNTs have been reported to partly include similar features to PA, such as Rosenthal fibers, oligodendroglial components, and hyalinized vessels [6]. The tumor of the present case may have been more similar to a RGNT rather than a PGNT because it exhibited PA-like features, a small amount of ganglion cells, and perivascular pseudorosettes that are unlikely in PGNTs [2]. The average age of onset in RGNT is reported to be higher than that in PGNT [4,6]. The present patient had another lesion in the fourth ventricle. However, we suggest that the lesion was a dissemination from the tumor of the lateral ventricle because of the tumor size and because of the patient's initial clinical symptoms. Thus, the fourth ventricle was not the primary site of the tumor in this case, which differs from RGNT cases.

The moderately high Ki-67 labeling index (LI) was another clue for the diagnosis of this tumor. The Ki-67 LI of ordinary glioneuronal tumors or central neurocytoma is less than 3%. The differential diagnoses were atypical central neurocytoma and PA with atypical features. Considering these histological variabilities of neuronal and glial component and the atypical histological findings, we finally diagnosed this case as an intraventricular tumor with atypical glioneuronal differentiation. According to Ishizawa et al. [7], there was a case of PGNT with proliferation of minigemistocytic component, which showed a high Ki-67 LI. This findings were considered to coincide with our case. However, it was reported that high Ki-67 LI in a case of PGNT was not nessesary associated with poor prognosis [8].

To the best of our knowledge, 11 cases of intracranial glioneuronal tumors (1 cases of PGNTs [9], 9 of malignant glioneuronal tumors [10] and 1 of RGNT [11]) presenting dissemination in the clinical course have been reported. There are also 3 cases of spinal cord glioneuronal tumors with neuropil-like islands presenting meningeal dissemination [12-14]. Javahery et al. [9] reported on a case of a 13-year-old girl with PGNT. She had a primary cystic lesion in the left frontal lobe, which was totally removed. However, 43 months after the initial surgery, the tumor relapsed adjacent to the primary site with dissemination to the pulvinar nucleus and the medial left thalamus, which disappeared with radiation therapy and concomitant temozolomide therapy. Varlet et al. [10] reported a summary of 9 cases with malignant glioneuronal tumors that exhibited dissemination with a median time to the event of 19 months after the initial treatment. Wang et al. [11] reported a case of 16-year-old girl with RGNT initially presenting intraventricular dissemination. She underwent a neuroendoscopic biopsy and postoperative radiotherapy. Accordingly, there were only 2 cases of intracranial glioneuronal tumors presenting with intraventricular dissemination at such an early time including our case. Attention may need to be paid to the dissemination in the case with glioneuronal tumor including the case of spinal cord origin.

Varlet et al. [10] reported that gross total resection of a malignant glioneuronal tumor was an independent and statistically significant prognostic factor. Additionally, they also stated that focal or craniospinal radiation therapy seemed neither to control local tumor growth nor to prevent dissemination of these tumors. Although our case underwent a partial resection of the tumor, the subsequent IMRT and adjuvant chemotherapy with temozolomide greatly reduced the residual tumors in the fourth ventricle and the anterior horn of the lateral ventricle. MRI with Gd demonstrated no recurrence of the residual small tumor 30 months after the surgery.

Following surgery, radiation therapy with concomitant administration of TMZ has recently become the standard therapy for malignant gliomas. As shown by the cases of Javahery et al. [9] and the present case, this therapy may also be effective for glioneuronal tumors.

## Conclusion

This is the rare case of glioneuronal tumor to present intraventricular dissemination at diagnosis, which have a possibility of transitional form between glial and neuronal components. The case was successfully treated wih a possible reductive surgery followed by IMRT with administarion of TMZ.

## List of abbreviations

HDR: Hasegawa's dementia scale; CT: computed tomography; MRI: magnetic resonance imaging; WI: weighted images; FLAIR: fluid-attenuated inversion recovery Gd: gadolinium; HE: hematoxylin-eosin; GFAP: glial fibrillary acidic protein; Syn: synaptophysin; TuJ1: tubulin, βIII isoform; Neu-N: neuronal nuclear antigen; MAP-2: microtubule associated protein-2; NSE: neuron-specific enolase; IMRT: intensity-modulated radiation therapy; PGNT: papillary glioneuronal tumors; PA: pilocytic astrocytoma; RGNT: Rosette-forming glioneuronal tumors of the fourth ventricle.

## Consent

Written informed consent was obtained from the patient for publication of this case report and accompanying images. A copy of the written consent is available for review by the Editor-in-Chief of this journal.

## Competing interests

The authors declare that they have no competing interests.

## Authors' contributions

HY was a major contributor in writing the manuscript. YH and NO participated in the pathological examination of the case. NN and TI participated in the surgery of the case. SY and JS participated in its design and coordination and helped to draft the manuscript. All authors have read and approved the final manuscript.

## Author's information

1 Department of Neurosurgery, Gifu University Graduate School of Medicine, Gifu, Japan

2 Pathology Division, Gifu University Hospital, Gifu, Japan

3 Chubu Medical Center for Prolonged Traumatic Brain Dysfunction, Department of Neurosurgery, Kizawa Memorial Hospital, Minokamo, Japan
